# HIV-1 infection among crack cocaine users in a region far from the epicenter of the HIV epidemic in Brazil: Prevalence and molecular characteristics

**DOI:** 10.1371/journal.pone.0199606

**Published:** 2018-07-17

**Authors:** Divânia Dias da Silva França, Nativa Helena Alves Del-Rios, Megmar Aparecida dos Santos Carneiro, Rafael Alves Guimarães, Karlla Antonieta Amorim Caetano, Monica Nogueira da Guarda Reis, Regina Maria Bringel Martins, Ana Rita Coimbra Motta-Castro, Mariane Martins de Araujo Stefani, Sheila Araujo Teles

**Affiliations:** 1 Department of Epidemiology, Municipal Secretary of Health of Goiânia, Goiás, Brazil; 2 Institute of Tropical Pathology and Public Health, Federal University of Goiás, Goiânia, Goiás, Brazil; 3 Faculty of Nursing, Federal University of Goiás, Goiânia, Goiás, Brazil; 4 Center of Biological and Health Sciences, Federal University of Mato Grosso do Sul, Campo Grande, Mato Grosso do Sul, Brazil; National and Kapodistrian University of Athens, GREECE

## Abstract

Brazil has the largest cocaine market in South America, and crack cocaine use is closely associated with HIV-1 infection. This study investigated the prevalence, risk factors, and HIV-1 subtypes, including recombinant forms and mutations associated with drug resistance, among crack cocaine users in Central-West Brazil. We recruited 600 crack cocaine users admitted to a referral hospital in Goiânia for psychiatric disorders. The participants were interviewed; blood samples were collected for anti-HIV-1/2 serological screening. HIV-1 *pol* gene sequences (entire protease [PR] and partial reverse transcriptase [RT]) were obtained from plasma RNA. HIV-1 subtypes, recombinant viruses, transmitted drug resistance (TDR), and secondary drug resistance mutations were investigated. The median participant age was 30 years (range, 18–68 years); most were male, single, unemployed, and of mixed races. Among them, 2.8% (17/600) were HIV-1 positive: 2.2% of men (11/507) and 6.5% of women (6/93). The main predictors of HIV-1 seropositivity were a sexual partner with HIV infection, irregular condom use, and previous homelessness. HIV-1 *pol* sequences (12/17) indicated the predominance of subtype B (n = 7), followed by recombinant forms F^PR^/B^RT^ (n = 1) and B^PR^/F^RT^ (n = 2) and subtypes F1 (n = 1) and C (n = 1). TDR prevalence was 58.3% (7/12). Isolates from two participants showed mutations associated with resistance to nucleoside reverse transcriptase inhibitors (NRTI) only (M41L, T125C, T125F, M184V), while an isolate from one patient who had received antiretroviral therapy (ART) since 2008 had a mutation associated with resistance to non-NRTI (G190S). Five isolates had secondary mutations to protease inhibitors (K20M, L10V, L33I, A71T, A71V). In conclusion, the findings of HIV-1 circulation, TDR to NRTI, and secondary mutations to protease inhibitors in ART-naïve crack cocaine users support the importance of monitoring this population in regions far from the epicenter of the HIV epidemic.

## Introduction

Unlike other regions worldwide, in South America the annual prevalence of cocaine use continues to rise, from 0.7% in 2010 (1.84 million users) to 1.2% in 2012 (3.34 million users), remaining at the same level in 2013. Furthermore, Brazil has been known to have the largest cocaine market in South America [1], and two large national surveys have estimated the prevalence of crack cocaine users to be 0.8%, representing 370,000 users [2, 3].

Crack cocaine is synthesized from cocaine hydrochloride, and its alkaline nature makes it suitable for smoking [1]. Its consumption has been associated with many psychiatric conditions, violence, and high rates of mortality [4–6]. Additionally, crack cocaine use has shown a high association with sexually transmitted infections (STI) due to the increased frequency of sexual risk behaviors [7, 8]. A close relationship has been described between crack cocaine use and HIV-1 infection, and high HIV-1 prevalence rates have been observed among crack cocaine users in Western countries [9–13]. Furthermore, crack cocaine seems to facilitate HIV disease progression [14].

Brazil is known to serve as a key strategic country for trafficking cocaine from Latin to North America and Europe [1]. In this regard, the central-west region (the states of Mato Grosso, Mato Grosso do Sul, and Goiás) is a recognized passageway for drug trafficking to the rest of the country and to foreign countries. According to a national household population survey, the central-west region currently shows the highest rates of use for both snorted and smoked crack cocaine and the highest prevalence of lifetime use for smoked cocaine [2].

A Brazilian survey estimated that 5% of crack cocaine users are infected with HIV, representing an 8-fold higher prevalence than estimated for the general Brazilian population (0.6%) [3, 15]. However, in a continental country like Brazil, with a wide socioeconomic diversity, pooled data may not be representative of the HIV epidemics in the diverse individual subregions. To understand these differences better, this study investigated the prevalence of and risk factors for HIV infection among crack cocaine users admitted to a referral hospital in Central-West Brazil for psychiatric disorders. In addition, we analyzed transmitted drug resistance (TDR) mutations and HIV-1 diversity in this vulnerable population.

## Materials and methods

This study was conducted among crack cocaine users admitted for detoxification at the Chemical Dependency Unit of the referral hospital for mental disorders in the city of Goiânia (with a population of 1,256,514), Central-West Brazil, from August 2012 to April 2013. The hospital covers 100% of all public hospitalizations related to drug abuse or illicit drug dependence in Goiânia.

The inclusion criteria for this study were crack cocaine use in the last 6 months, age ≥18 years, and being on treatment for drug abuse. The exclusion criterion was showing of altered behavior that made it impossible for the subject to answer clearly the questions or for blood samples to be collected. Altered behaviors included anger manifestations, and verbal and physical threats.

A total of 1,305 patients were admitted to the Chemical Dependency Unit. Among them, 959 (73.5%) had a history of crack cocaine use, 693 were identified as eligible, and 600 were enrolled in the study (86.6%). All participants were previously informed about the objectives of the study, and provided written informed consent. The interviews were face-to-face in a private setting at the hospital. We modified and used with permission a questionnaire previously used in the National Research Study on Crack Use in Brazil [3]. Data on sociodemographic characteristics, risk behaviors, and drug use profile were collected. After the interview, blood samples were collected from all participants for HIV serology and molecular analysis.

All sera specimens were tested for the presence of anti-HIV-1/2 antibodies using an enzyme-linked immunosorbent assay (ELISA) (Wiener Lab, Argentina). Positive and borderline results were confirmed by a Western blot (NEW LAV BLOT I, Bio-Rad, France).

### HIV-1 genetic characterization

HIV-1 RNA extracted from plasma [(QIAamp Viral RNA Mini Kit (Qiagen GmbH, Hilden, Germany) was used for reverse transcription and used as the target for a nested PCR of the pol region. The 849 pb pol fragment sequenced included the entire protease (PR) region (99 bp; HXB2, 2253–2549 positions) and approximately 750 bp of the reverse transcriptase (RT) fragment (HXB2, 2550–3299 positions) which were amplified employing K1/K2 (external primers) and DP10/F2 (internal primers) as described previously by Cardoso et al [16]. After purification of amplicons (QIAquick PCR Purification Kit/QIAGEN, Qiagen GmbH, Hilden, Germany), genomic sequencing was performed (GeneAmp PCR System 9700, Applied Biosystems Inc, Foster City, CA, USA). The HIV-1 subtypes were identified by REGA genotyping tool version 2.0 and by phylogenetic inference using reference sequences obtained from Los Alamos HIV Database (http://hiv_web.lanl.gov). Trees for phylogenetic analysis were generated by the neighbor joining method under Kimura’s two-parameter correction model using MEGA version 5 software. Bootstrap values (1,000 replicates) above 70% were considered significant. SimPlot 3.5.1 was used to analyze intersubtype recombination in sequences with discordant subtypes in the PR and RT fragments.

The existence of possible transmission clusters, were investigated by phylogenetic trees (Neighbor- Joining, Kimura two parameters) setting a minimum bootstrap value of 70%. Our study sequences were analysed with four different sets of sequences including HIV-1 subtype B, F1, C and BF. Sequences herein identified were submitted to a Basic Local Alignment Search Tool, BLAST, Los Alamos available at (https://www.hiv.lanl.gov/content/sequence/BASIC_BLAST/basic_blast.html)) and BLAST searches were performed in order to recover other sequences with high similarity (>95%) and possibly belonging to the same transmission clusters. Subtype B sequences from the same endemic region (Goias State, central west Brazil) deposited at the Genbak between 2003–2013 were also retrieved to construct a phylogenetic tree with reference sequences of HIV-1 subtypes A-D, F-H, J e K.

Transmitted drug resistance (TDR) rate among ARV-naïve patients was determined by the Calibrated Population Resistance tool (Stanford Surveillance Drug Resistance Mutations-SDRM). Secondary drug resistance mutations and resistance profiles were defined by the Stanford HIV Drug Resistance Database. The ARV mutation susceptibility profile was analyzed by the Stanford HIV Drug Resistance Database.

### Data analysis

Prevalence was calculated at 95% confidence levels. A chi-square test, Fisher’s exact test (for categorical variables), and Student’s t-test (for quantitative variables) were used to compare variables and determine *p* values (two-tailed). Odds ratios and 95% confidence intervals (CI) were used as measures of the strength of association between HIV positivity (outcome) and independent variables. Variables associated with the outcome, with *p* values <0.10, were entered into a backward stepwise logistic regression model. Statistical analyses were performed using SPSS Statistics for Windows, version 17.0 (IBM Corp., Armonk, NY, USA). S1 File presents the database and dictionary of variables.

### Ethical issues

This study was approved by the local review board (Committee on Ethics in Human Research of Hospital das Clínicas, Universidade Federal de Goiás, Brazil). Informed consent was obtained from the participants.

## Results

The median age of the participants was 30 years (range, 18–68 years). The majority (84.5%) were male and self-reported as nonwhites (75.8%). Half of crack cocaine users had less than a fundamental level of education. In this group, 26% reported being officially employed, and 20.3% reported being previously homeless. Of the total, 2.8% (17/600) were HIV-1 positive (95% CI, 1.8–4.5), ranging from 2.2% (11/507) (95% CI, 1.2–3.8) in men to 6.5% (6/93) (95% CI, 3.0–13.4) in women. Eight out of the 17 had prior knowledge of their HIV serological status.

Tables 1 and 2 present the bivariate and multivariate logistic regression analysis results, respectively, for risk factors. In the bivariate analysis, the female sex, intensive crack cocaine use (>10 rocks/day), having more than two sexual partners in the last 6 months, previous STI, sexual intercourse with people living with HIV, unprotected sex, and living on the streets in the last 6 months were significantly associated with HIV positivity (*p* < 0.05).

**Table 1:** Demographic and social characteristics and sexual and drug consumption behaviors among 600 in-treatment crack cocaine users.

Variable	n	%	HIV				
			Positive	%	Negative	%	*p*
**Demographic and social characteristics**							
Age group >30 years (median: 30; range: 18–68)	273	45.5	09	3.3	264	96.7	0.53
Gender							
Male	507	84.5	11	2.2	496	97.8	**0.02**
Female	93	15.5	6	6.5	87	93.5	
Race (self-declared)^¥^							
White	145	24.2	03	2.1	142	97.9	0.89
Black	46	7.7	01	2.2	45	97.8	
Mixed-race Brazilian	369	61.5	12	3.3	357	96.7	
Asian	40	6.7	1	2.5	39	97.5	
Legal employment	156	26.0	03	1.9	153	98.1	0.58
Education (years)	302	50.3	10	3.3	292	96.7	0.48
0–4	64	10.7	2	3.1	62	96.9	
5–9	309	51.5	6	1.9	303	98.1	
10–12	180	30.0	7	3.9	173	96.1	
>12	47	7.8	2	4.3	45	95.7	
History of living on the streets*	122	20.3	07	5.7	115	94.3	**0.03**
Previous arrest	295	49.2	10	3.4	285	96.6	0.42
**Sexual risk**							
Exchange sex for money/drugs*^a^	114	19.0	04	3.5	110	96.5	0.81
More than 2 sexual partners*	243	40.5	11	4.5	232	95.5	**0.04**
Sexual intercourse with PLWHIV^b^	24	4.0	05	20.8	19	79.2	**<0.001**
Unprotected sex*^c^	190	31.7	11	5.8	179	94.2	**0.01**
Previous STI ^d^	154	25.7	10	6.5	144	93.5	**<0.01**
Homosexual/bisexual^e^	41	6.8	03	7.3	38	92.7	**0.13**
Sexual violence	71	11.8	01	1.4	70	98.6	0.71
Testing for HIV^f^	343	57.2	13	3.8	330	96.2	0.14
**Drug consumption behavior**							
Smoking crack cocaine from improvised pipes	416	69.3	11	2.6	405	97.4	0.67
Sharing pipes/improvised pipes^g^	438	73.0	15	3.4	423	96.6	0.22
Crack cocaine use >48 months	265	44.2	07	2.6	258	97.4	0.80
Intensive crack cocaine use (>10 rocks/day)^h^	181	30.2	09	5.0	172	95.0	**0.03**
Presence of oral sores	189	31.5	08	4.2	181	95.8	0.16
Previous injection drug use (illicit drugs)	57	9.5	03	5.3	54	94.7	0.21

HIV, human immunodeficiency virus; PLWHIV, people living with HIV; STI, sexually transmitted infections.

^¥^ According to the definition of the *Instituto Brasileiro de Geografia e Estatística—IBGE*

* In the last 180 days.

^a^ 62 missing observations.

^b^ 80 missing observations.

^c^ 67 missing observations.

^d^12 missing observations.

^e^ 57 missing observations.

^f^ 4 missing observations.

^g^ 38 missing observations.

^h^ 32 missing observations.

**Table 2:** Multivariate analysis of factors associated with HIV infection among 600 in-treatment crack cocaine Users.

Variable	OR (95% CI)	Adjusted OR (95% CI)^a^	*p*
Sexual intercourse with PLWHIV	14.2 (4.3–46.6)	13.1 (3.2–54.1)	**<0.001**
Previous STI	4.2 (1.6–11.3)	3.2 (1.0–10.4)	0.06
Unprotected sex	3.4 (1.3–9.5)	2.8 (0.9–9.3)	0.08
Living on the streets	2.8 (1.1–7.6)	4.2 (1.1–15.4)	0.03
Intensive crack cocaine use (>10 rocks/day)	2.8 (1.0–7.7)	2.8 (0.9–8.9)	0.08

OR, odds ratio; CI, confidence interval; HIV, human immunodeficiency virus; PLWHIV, people living with HIV; STI, sexually transmitted infections.

^a^ Adjusted OR for the following variables: age, sex, previous HIV testing, unprotected sex, more than 2 sexual partners, previous STI, living on the streets, and intensive crack cocaine use.

These variables were entered into a multivariate logistic regression model to identify independent predictors of HIV-1 infection. From this model, participants who reported having sex with an HIV-infected individual increased their chance of being infected with HIV-1 by 13.1 times (95% CI, 3.2–54.1). Individuals with a history of living on the streets had a 4.2-fold (95% CI, 1.1–15.4) greater chance of being HIV-1 positive compared with those without. In addition, two variables showed a marginal association with HIV-1 infection: unprotected sex (adjusted OR, 2.8; 95% CI, 0.9–9.3) and >10 stones or crack cocaine portions per day (adjusted OR, 2.8; 95% CI, 0.9–8.9) (Table 2).

**Fig 1:**
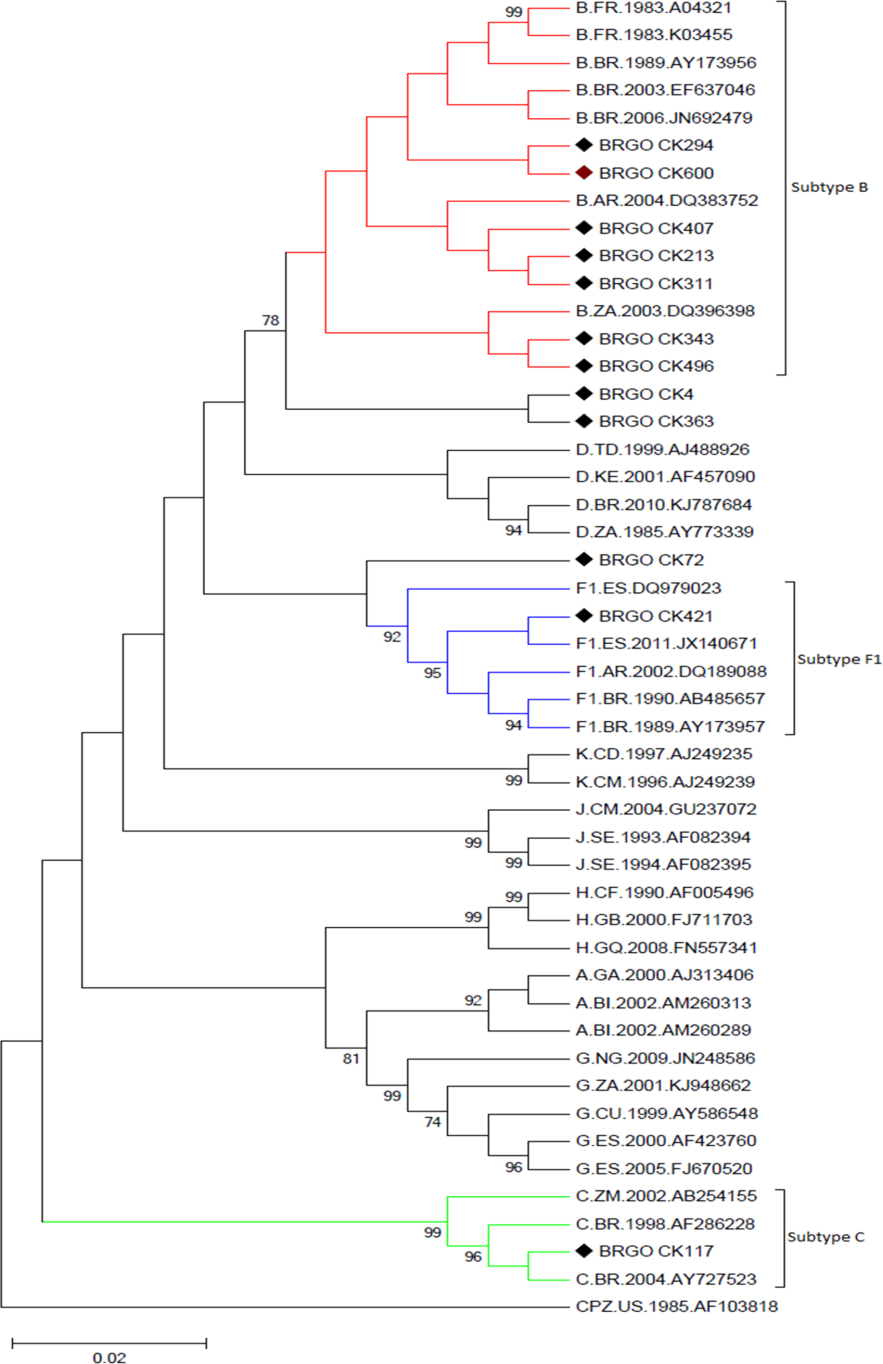
Phylogenetic analyses of HIV-1 in protease (PR) and reverse transcriptase (RT) fragments among crack cocaine users in Goiânia. The isolates characterized in the study are given in bold, and the clusters are marked with the symbol ♦. Using Molecular Evolutionary Genetics Analysis software version 5.05, trees were constructed by the neighbor-joining method with Kimura’s two-parameter distance correction model (bootstrap values >70% were considered significant).

In 12 out of 17 crack cocaine users (70.6%), sequences of the PR and RT regions of the HIV-1 *pol* gene were generated and analyzed. Seven of the 12 (58.3%) were infected with subtype B, one (8.3%) with subtype F1, and one (8.3%) with subtype C. Three participants (25%) had BF1 recombinant forms (Fig 1): one with F^PR^/B^RT^ and two with B^PR^/F^RT^.

Possible transmission clusters of subtype B clade were investigated constructing phylogenetic trees with four different datasets. Phylogenetic analyses were performed using 199 subtype B sequences from the same endemic region (S1 Fig), BLAST selected sequences including 52 subtype B sequences with high similarity (above 95%) (S2 Fig), a third dataset including 40 subtype B sequences retrieved from the Genbank which were collected among 10 Brazilian intravenous drug users [17] and 30 from prisoners collected in the same geographical region [18] (S3 Fig). The fourth data set included 95 BLAST selected isolates with high similarity (above 95%) with subtype B, C, F1 and BF from our study (S4 Fig). None of the phylogenetic analyses performed indicated the existence of transmission clusters among our samples defined by clustering with bootstrap >70%.

Two crack users harbored HIV-1 isolates that presented mutations to reverse transcriptase inhibitors/RTI. One (male; age 37) patient previously diagnosed and under antiretroviral treatment/ ART since 2008, had dual class secondary resistance. This patient’s isolate (subtype B, ID# BRGO_CK343) had mutations associated with nucleoside reverse transcriptase inhibitors/NRTI (T215F and M184V) and non-nucleoside reverse transcriptase inhibitors/NNRTI (G190S). Transmitted drug resistance was identified in one (age 29) ARV-naive female crack user that harbored virus with mutations to NRTI (M41L, T125C). No major mutation to PI was identified; however, five isolates presented minor PI mutations (K20M: BRGO_CK4, subtype B; A71V: BRGO_CK600, subtype B; L10V: BRGO_CK496, subtype B; A71T and L33I: BRGO_CK311, subtype B; L10V: BRGO_CK117, subtype C). Only one of them had experienced ARV therapy (BRGO_CK600).

## Discussion

Crack cocaine users represent a hard-to-reach population with important social and public health implications. They usually live on the margins of the society and are highly exposed to and involved in illicit events often associated with violence and crime [19, 20]. These characteristics are major barriers to reaching this population in their environment. Therefore, due to safety and logistical issues, convenience sampling can be a practical method of studying this population. Though the results may not be representative of the population as a whole, the strategy can offer valuable information for understanding the dynamics of infection in specific hard-to-reach populations.

We investigated 600 crack cocaine users admitted to a referral hospital that attends to patients from the public health system in the city of Goiânia. In this population, some social characteristics such as education and race/ethnicity suggest better socioeconomic conditions compared with being recruited/living on the streets [13, 21, 22], although they have common risk behaviors.

In 2012, the National Research Study on Crack Use in Brazil [3], which used time-location sampling, estimated a global HIV-1 prevalence of 5.0% (95% CI, 3.7–6.6) [23] in this population. This rate is higher than that found among the in-treatment crack cocaine users investigated in this study (2.8%; 95% CI, 1.8–4.5), although there is an overlap of CIs. On the other hand, this prevalence is almost 5-fold greater than the estimated prevalence of HIV-1 infection in the general Brazilian population (0.6%) and is within the large prevalence range observed among crack cocaine users in the country. For example, in community-based samples, rates of 1.6% (95% CI, 0.2–5.7) and 11.2% (95% CI, 4.2–18.2) were estimated in 125 women and 79 mixed-sex users from Salvador, northeastern Brazil, respectively [22, 24]. In relation to other studies on crack cocaine users in Brazil, variable HIV-1 infection rates were reported: 37% (95% CI, 26.8–48.5) in Porto Alegre City, southern region [25], and 4.0% (95% CI, 0.0–8.0) in Rio de Janeiro, southeast region [13]. Variable rates were also observed among in-treatment crack cocaine users in the same regions. While a study in Rio de Janeiro [13] did not find any case of HIV infection in 30 crack cocaine users, another investigation in the city of Campinas, in the state of São Paulo [19], reported the prevalence of HIV-1 infection to be 11% (95% CI, 7.7–14.3) among 132 users. For any event with a small prevalence, a small sample size presents an important limitation, which was not the case in our study.

Since only 15.5% (93/600) of participants were women, they were disproportionally affected by HIV-1 compared with men (6.5% vs. 2.2%). In general, female crack cocaine users reported a higher sexual risk behavior than men, in particular, exchanging sex for drugs/money. In addition, they are highly vulnerable to sexual violence and abuse, both of which have been associated with HIV infection [21, 26, 27]. In fact, in this study, 51.1% of women reported exchanging sex for drugs/money (vs. 15.3% of men), and 37.1% suffered sexual violence (vs. 7.1% of men) (data not shown).

Our findings support the knowledge that sexual transmission represents the major mode of HIV dissemination among crack cocaine users [28–30]. Sex with an HIV-infected partner was the strongest predictor of HIV-1 infection. In addition, though the association with unprotected sex and previous STI weakens after controlling for confounding factors, they retain a marginal association, suggesting lack of association due to the sample size.

Crack cocaine is a stimulant drug that reduces the perception of risk, self-care, and social values. The user is usually more concerned about getting the pleasures related to the effects of the drug than adopting safety measures to preserve their physical and mental integrity. Therefore, neglecting condom use during sexual intercourse is a common practice among them [21]. The present findings support this assumption; 190 crack cocaine users reported having unprotected sex, and this behavior was marginally associated with HIV-1 infection.

Many crack cocaine users are socioeconomically marginalized, and a large proportion live on the streets or in unstable housing conditions [21, 31]. In this study, one out of five crack cocaine users reported living on the streets within the last 6 months. Thus, this variable was considered a predictor of HIV-1 positivity, supporting the health and social harms related to crack cocaine use.

As previously reported, our study found a higher prevalence of HIV-1 among individuals who reported more intensive crack cocaine use (>10 rocks/day), supporting the notion that a high level of dependence on illegal drugs has been associated with a higher prevalence of risk behaviors [32].

This study found the predominance of subtype B, followed by recombinant forms (BF1) and subtypes C and F. This distribution is similar to that found in Brazil as a whole [33], except in the southern region where subtype C is predominant. It is noteworthy that the detection of HIV-1 subtype C among crack cocaine users in Goiânia supports the circulation of this isolate among drug users in the central-west region. HIV-1 subtype C has rarely been found among drug users outside the southern region. However, in a recent publication [17] about a survey of HIV-1 among drug users from eight Brazilian cities outside the southern region, subtype C was identified only in Campo Grande and Brasilia, both cities in the central-west region.

We have used molecular data analysis as a proxy for an epidemiological relationship and the identification of a possible molecular cluster, defined as a group of persons with diagnosed HIV infection who have genetically similar HIV strains which suggest transmission [34]. In our analyses, no subtype B transmission cluster was identified using phylogenetic analyses with different subtype B sequences datasets, including regional subtype B sequences, a BLAST selected dataset of sequences with high similarity with our sequences and sequences from Brazilian intravenous drug users and local prisoners. HIV-1 is a rapidly evolving pathogen and transmission clusters can be traced by its genome providing important information regarding transmission dynamics and epidemiology. The dynamics of HIV-1 transmission among high risk populations, such as crack users, can contribute to design improved prevention and intervention strategies [34]. Usually HIV-1 transmission chains can be identified based on sequence similarity determined directly from sequence alignment or indirectly by phylogenetic inference. Phylogenetic analyses have been effectively used to identify and analyze HIV-1 transmission clusters and together with epidemiological and clinical information they can be of public health importance providing relevant information about virus dissemination among groups with different risk factors [35–40]. Our phylogenetic analyses did not indicate the existence of transmission clusters within the group of crack addicted individuals infected with HIV-1.

There are scanty data regarding ART resistance mutations among illicit drug users in Brazil [17]. This investigation found transmitted drug resistance (TDR) to NRTI in one ART-naive crack user (ID# BRGO_CK213). This patient’s virus had M41L mutation which is a TAM that usually occurs with T215Y conferring high-level resistance to AZT and d4T and intermediate-level resistance to ddI, ABC and TDF. T215C is considered a revertant mutation that do not reduce NRTI susceptibility but indicate that this virus population once contained T215Y/F mutations. This mutation suggests that the patient may have once harbored a majority virus population with T215Y/F. The detection of ARV-naive crack users already bearing TDR mutations to NRTI constitutes a major concern in terms of dissemination of drug resistant viruses in regions far from the epicenter of HIV epidemic.

Four of five ART-naive crack users showed minor mutations to PI (polymorphisms) which can lead to phenotypic resistance to PIs in future ART-treatment since the development of PI resistance generally requires the accumulation of both major and minor mutations in the protease.

This study has some limitations. The generalizability of our data is limited because the participants were from only one drug dependence service, though it covers 100% of all public hospitalizations related to drug abuse or illicit drug dependence in the city. Additionally, self-reports of sexual behaviors may overestimate or underestimate the chances of HIV positivity. Further, our small sample size has probably reduced the chances of identifying possible transmission clusters. Also, the relatively long terminal branches of our sequences shown in the phylogenetic trees indicate higher diversity suggesting that these individuals were probably infected many years before being sampled. HIV-1 accumulates genetic changes over time so that shortly after transmission of HIV between two individuals, there will be very little or no genetic sequence diversity between transmitting and recipient strains, favoring the identification of transmission clusters. Small sample size and probably long time since infection may have hampered the identification of transmission clusters in our study.

## Conclusions

To the best of our knowledge, this is the first study regarding the epidemiology of HIV among crack cocaine users in Goiânia, Central-West Brazil, providing insights into HIV-1 circulation and molecular characteristics and facilitating target interventions aimed at preventing HIV in this high-risk population. The findings of TDR to NRTI and secondary mutations to PI in ART-naïve crack cocaine users support the importance of monitoring this population in regions far from the epicenter of the HIV epidemic.

## Supporting information

S1 Fig Phylogenetic analysis of HIV-1 protease and reverse transcriptase fragments to investigate transmission clusters of subtype B clade among crack cocaine users in Goiania, Brazil. Subtype B study sequences and 199 subtype B sequences from the same geographic region, retrieved from the GenBank were used. The phylogenetic tree was generated using neighbor-joining under Kimura's two-parameter correction model (MEGA version 5 software) and transmission clusters were defined by bootstrap >70%.
(TIFF)

S2 Fig Phylogenetic analysis of HIV-1 protease and reverse transcriptase fragments to investigate transmission clusters of subtype B clade among crack cocaine users in Goiania, Brazil. Subtype B study sequences and 52 BLAST selected subtype B sequences with similarity above 95%, retrieved from the GenBank were used. The phylogenetic tree was generated using neighbor-joining under Kimura's two-parameter correction model (MEGA version 5 software) and transmission clusters were defined by bootstrap >70%.
(TIF)

S3 Fig Phylogenetic analysis of HIV-1 protease and reverse transcriptase fragments to investigate transmission clusters of subtype B clade among crack cocaine users in Goiania, Brazil. Subtype B study sequences and 99 subtype B sequences of prisoners from the same geographic region and from Brazilian intravenous drug users, retrieved from the GenBank were used. The phylogenetic tree was generated using neighbor-joining under Kimura's two-parameter correction model (MEGA version 5 software) and transmission clusters were defined by bootstrap >70%.
(TIF)

S4 Fig Phylogenetic analysis of HIV-1 protease and reverse transcriptase fragments to investigate transmission clusters among crack cocaine users in Goiania, Brazil. Study sequences and 95 BLAST selected subtype B, C, F1 and BF sequences with similarity above 95%, retrieved from the GenBank were used. The phylogenetic tree was generated using neighbor-joining under Kimura's two-parameter correction model (MEGA version 5 software) and transmission clusters were defined by bootstrap >70%.
(TIF)

S1 File Database and variable dictionary. (XLSX)
