# Supplementary figures and images for: HIV-1 infection among crack cocaine users in a region far from the epicenter of the HIV epidemic in Brazil: Prevalence and molecular characteristics
Source: PLoS One. 2018 Jul 17;13(7):e0199606. doi: 10.1371/journal.pone.0199606 (PMC6049907; doi:10.1371/journal.pone.0199606)

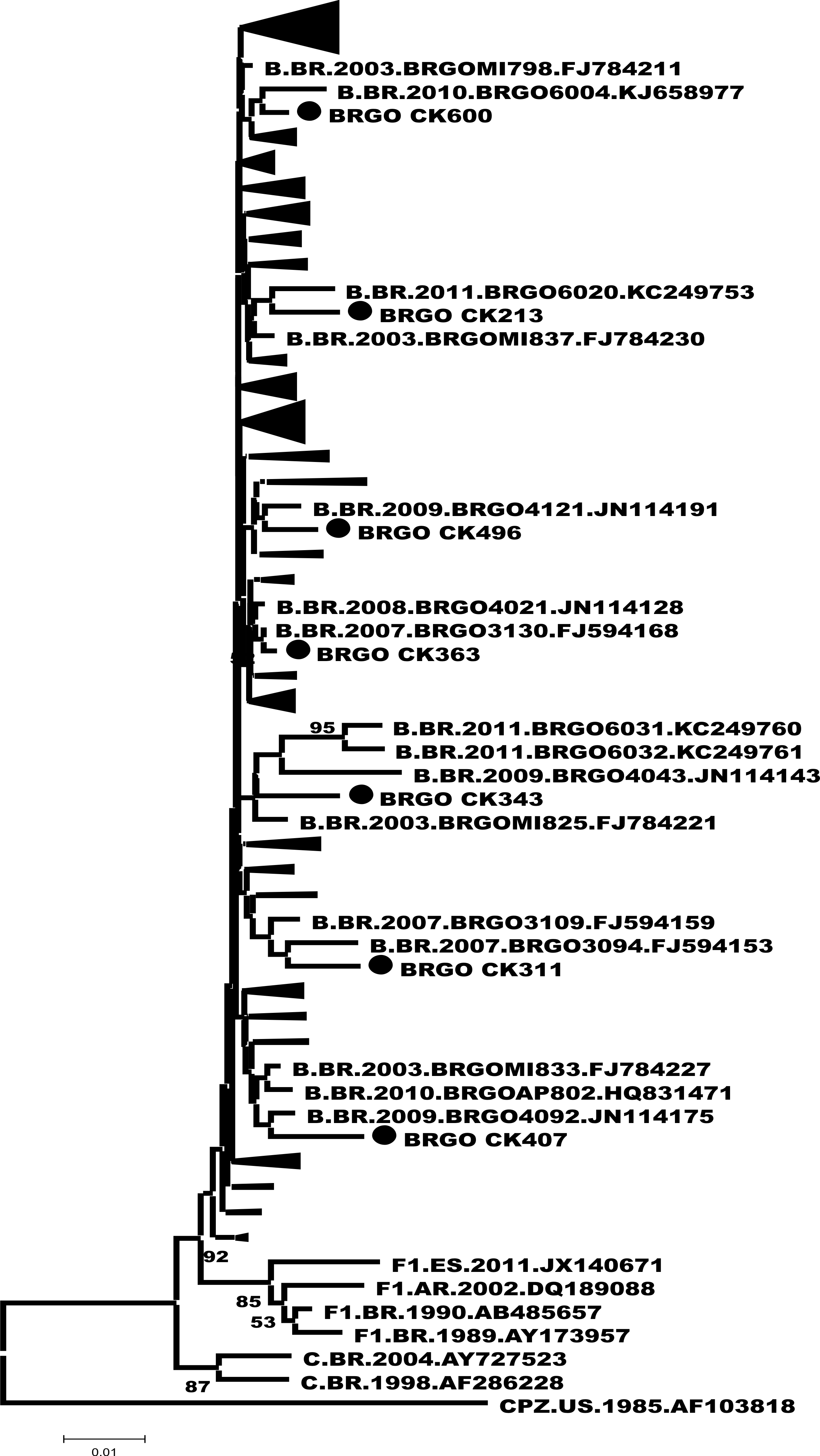

Supplement: S1 Fig — Subtype B study sequences and 199 subtype B sequences from the same geographic region, retrieved from the GenBank were used. The phylogenetic tree was generated using neighbor-joining under Kimura's two-parameter correction model (MEGA version 5 software) and transmission clusters were defined by bootstrap >70%. (TIFF) [file pone.0199606.s001.tiff]

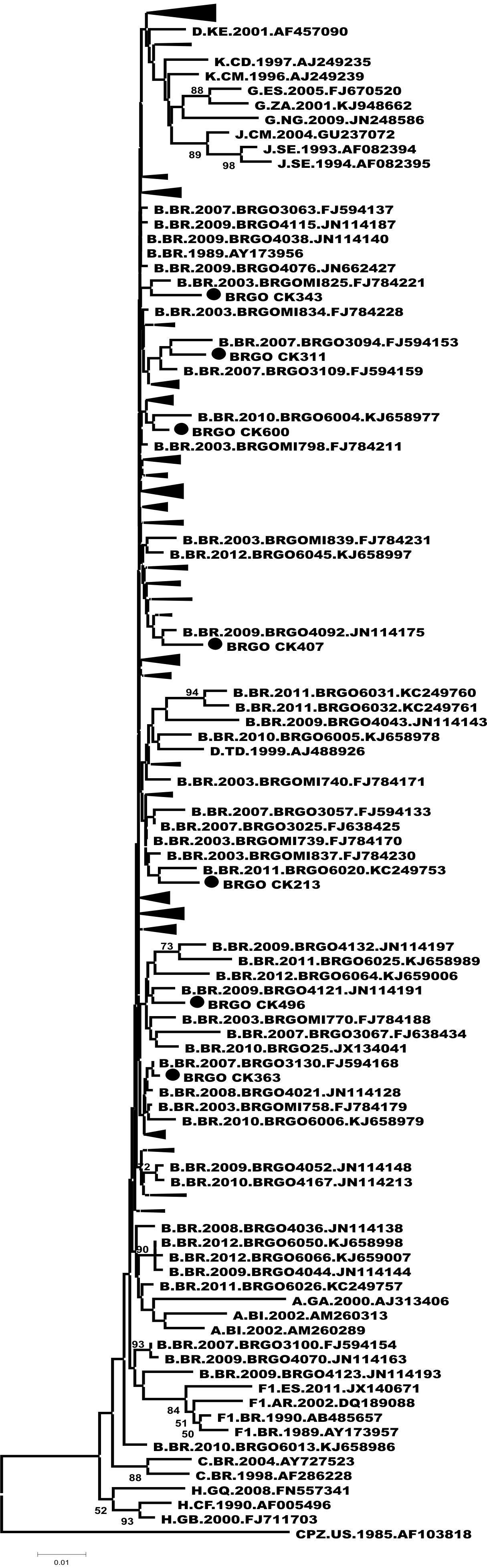

Supplement: S2 Fig — Subtype B study sequences and 52 BLAST selected subtype B sequences with similarity above 95%, retrieved from the GenBank were used. The phylogenetic tree was generated using neighbor-joining under Kimura's two-parameter correction model (MEGA version 5 software) and transmission clusters were defined by bootstrap >70%. (TIF) [file pone.0199606.s002.TIF]

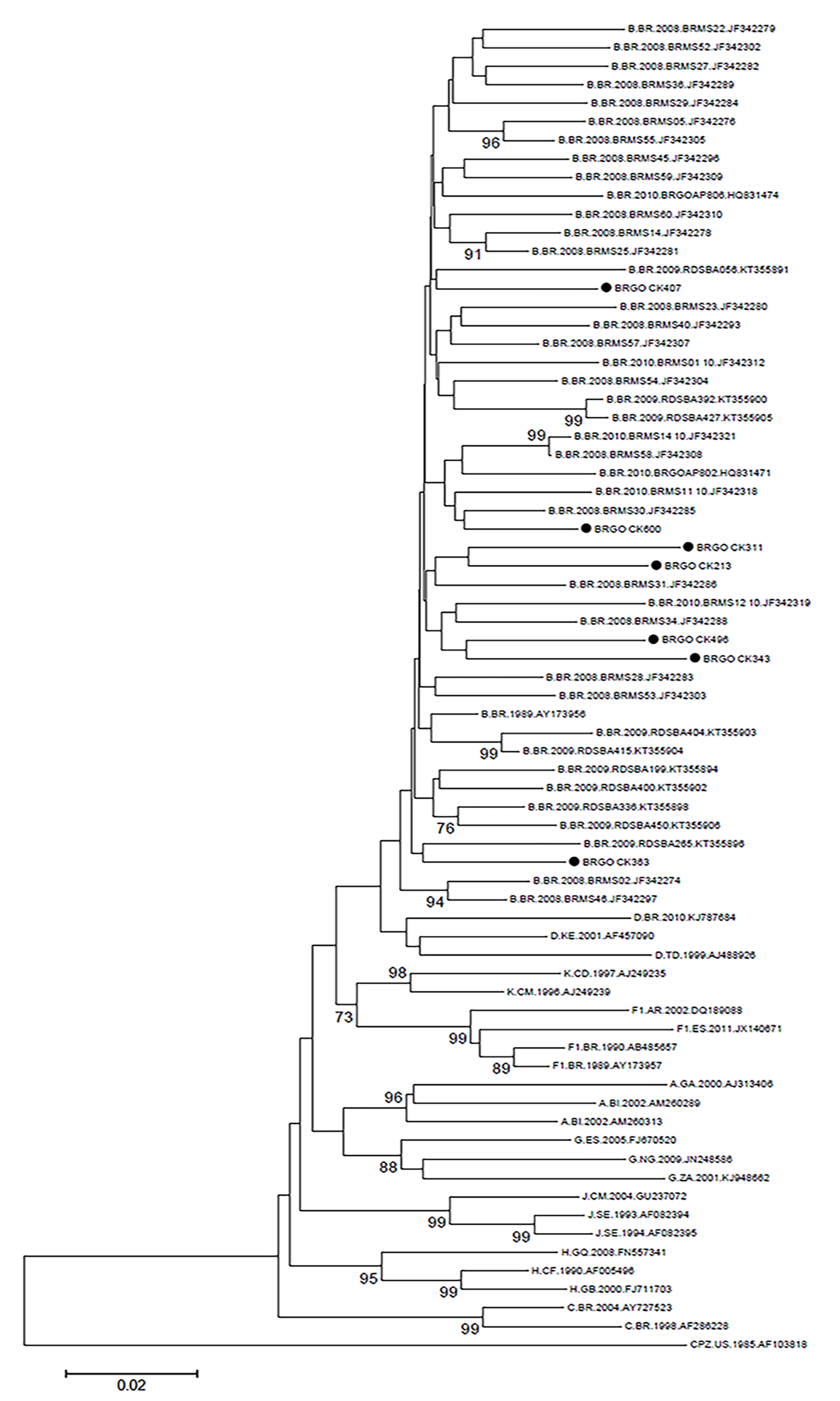

Supplement: S3 Fig — Subtype B study sequences and 99 subtype B sequences of prisoners from the same geographic region and from Brazilian intravenous drug users, retrieved from the GenBank were used. The phylogenetic tree was generated using neighbor-joining under Kimura's two-parameter correction model (MEGA version 5 software) and transmission clusters were defined by bootstrap >70%. (TIF) [file pone.0199606.s003.tif]

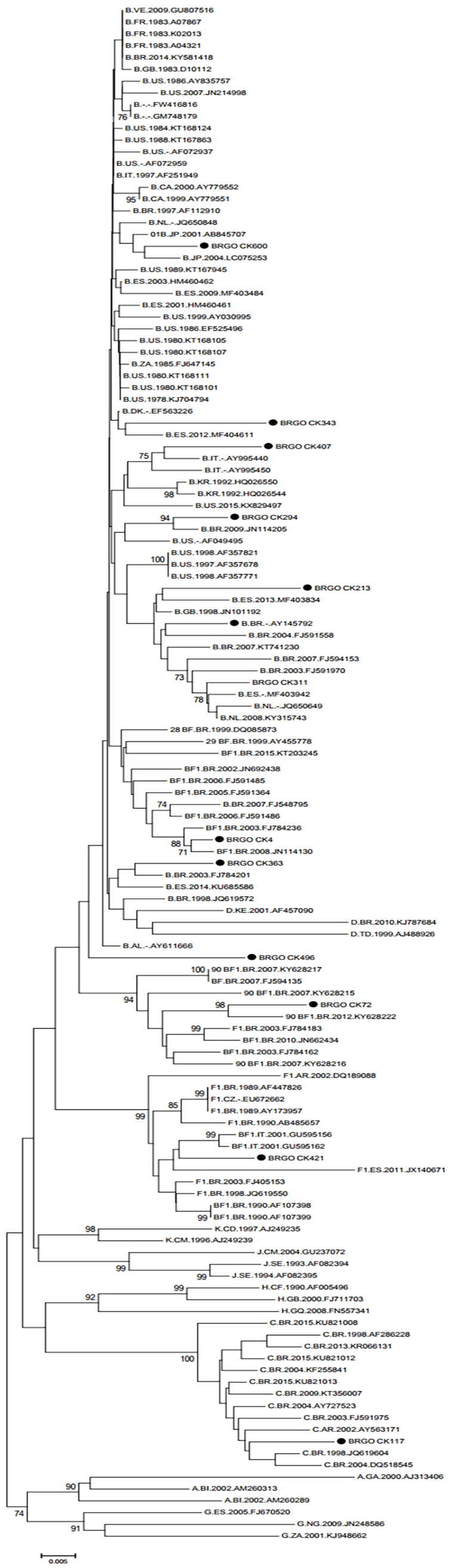

Supplement: S4 Fig — Study sequences and 95 BLAST selected subtype B, C, F1 and BF sequences with similarity above 95%, retrieved from the GenBank were used. The phylogenetic tree was generated using neighbor-joining under Kimura's two-parameter correction model (MEGA version 5 software) and transmission clusters were defined by bootstrap >70%. (TIF) [file pone.0199606.s004.tif]
